# A Global Cypher: The Role of Hip Hop in Cultural Identity Construction and Navigation for Southeast Asian American Youth

**DOI:** 10.1002/cad.20279

**Published:** 2019-03-19

**Authors:** Jacqueline Nguyen, Gail M. Ferguson

**Affiliations:** ^1^ University of Wisconsin‐Milwaukee; ^2^ University of Illinois at Urbana‐Champaign

## Abstract

Southeast Asian American (SEAA) adolescents and emerging adults navigate a multicultural, global world by utilizing cultural variability to play up and play down three cultural identities: their Asian/Asian American heritage culture, the White dominant culture in which they live, and a hip hop cultural identity. The latter is a unique cultural identity rooted in the global phenomenon of hip hop that includes dance, art, and music as well as resistance to the dominant, mainstream culture. Hip hop is a meaningful cultural identity for SEAA youth because it is a cultural identity transcendent of race/ethnicity, a means toward relational and identity harmony, a form of resistance, and because it facilitates belongingness to a local and a global community.

Hip hop refers to both a musical style/genre and a subculture originating in the 1970s out of the Bronx, New York. It has roots in African American, Jamaican American, and Latino American communities and was borne out of resistance to dominant mainstream, predominantly White U.S. culture. Whereas it was originally “a set of cultural practices rooted in multicultural urban neighborhoods throughout the U.S.” (Dao, 2014, p. 97), its mainstream and commercial appeal have spread globally and according to analyses of the music streaming service Spotify, hip hop is by far, the most listened‐to genre across the world (Hooton, 2015, July 14). Thus, hip hop is now a multicultural, global phenomenon that includes a range of activities including dance, music (i.e., rapping/MCing), DJing, and graffiti (Dimitriadis, 2009), and youth today engage in hip hop to develop pluralistic, hybrid identities worldwide. Indeed, the language of such global connectedness is built into hip hop with the term *cypher* referring to gatherings in which participants physically or musically perform together to shape the culture. In breakdancing, it refers to a practice in which a crowd of spectators form in a circle and participants take turns performing and sharing their skills with others. In today's digital age, the *global cypher* has grown as a result of online sharing of performances and music.

Despite this mainstream popularity, the core ideologies of hip hop remain tenaciously preserved by participants. In particular, by youth of color who regard it as a physical and psychological multicultural cultural space to foster political engagement, resist of racial hierarchies, and challenge of stereotypes crafted about ethnic minority youth by the dominant culture (Langnes & Fasting, 2014). Additionally, youth around the world increasingly use hip hop for a sense of identity agency, to expand their identity possibilities and critique/resist the constraints of monolothic identity choices dictated by their societies (Huq, 2006). Among many examples, hip hop has been used by Latinx youth to “critique their racialized identities in schools and society” (Pulido, 2009), by Aboriginal Australians to politicize and modernize a transnational Black identity (Morgan & Warren, 2011), by marginalized Maori and Pacific Islander youth as an identity marker (Kopytko, 1986), by Mongolian youth seeking to distinguish their generation from older, socialist‐era adults (Marsh, 2010), and by Chinese youth to showcase their individual, hybridized identities in resistance to a national culture that suppresses individualism (Barrett, 2012).

Hip hop has a historic and quintessentially global place in the zeitgeist of cultural identities (Alim, Ibrahim, & Pennycook, 2008) and is a uniquely flexible cultural identity. A hip hop cultural identity is malleable and can be played up/played down through a process called cultural variability as youth adapt to the sociocultural and interpersonal demands of adolescence/emerging adulthood. Cultural variability is a newly introduced identity development process in which individuals play up/play down aspects of their cultural identity on a daily basis (Ferguson, Nguyen, Iturbide, & Giray, 2017), providing an ideal concept through which to understand the function of hip hop in adolescent and emerging adult identity. This article examines hip hop as a third cultural identity for Southeast Asian American (SEAA) youth—one with unique capabilities for cultural variability—and how these youths’ engagement in hip hop is a result of, and a contribution to, a global hip hop identity and movement.

## Hip Hop as a Third Cultural Identity

Like many of their peers in today's globalized society, SEAA youth experience difficulty fitting neatly into the two prevailing identity categories available to them in U.S. society: (1) that of the White dominant culture to which SEAA youth are exposed on a daily basis by virtue of living in the United States, and (2) an Asian identity rooted in their home culture and/or an Asian American identity dependent on their perspectives about the distinction between “Asian” and “Asian American” (Lee & Zhou, 2004). This is an oversimplified binary (Ngo, 2008; Hickman, 2017). In actuality, SEAA youth struggle to find cultural identity amidst several sets of expectations: parental (gendered) beliefs about what it means to be a “good” or “bad” child (i.e., Xiong, Eliason, Detzner, & Cleveland, 2005), societal stereotypes due to both the “model minority” myth and the “perpetual foreigner” belief (Poon et al., 2015; Said, 1979), and evolving definitions of Asian immigrant, Asian American, and mainstream U.S. cultures (Crane, 2016; Mistry et al., 2016).

Given these nuanced complexities driven in part by globalization, it is increasingly common for youth to form identities that are rooted in multiple cultural traditions (Jensen, 2003). This is consistent with the tridimensional acculturation theory for individuals who are navigating three cultures: their heritage culture, the mainstream culture, and a societal subculture (Ferguson, Bornstein, & Pottinger, 2012). Hip hop is one such tricultural identity and provides opportunity to navigate and resist identity challenges (Chan, 2016). In fact, some SEAA youth have referred to themselves as “pencils” due to their self‐proclaimed identity as “yellow on the outside, White in the middle, and Black at the core” due to their engagement with hip hop culture (Nguyen, 2013). Hip hop helps SEAA youth develop a local identity while connecting to a broader global community.

## Hip Hop as “Glocal” and Asian

Hip hop is a global tool that is transcendent of any one particular cultural group or identity (Condry, 2007) yet simultaneously flexible enough to be adapted to youth in situ and reinterpreted for the local context in which it is used—thereby deeming it a “glocal” phenomenon (Gadet, 2015; Motley & Henderson, 2008). As such, participants can engage in local hip hop culture while feeling broadly connected to a global culture. Asian and Asian American youth have been at forefront of hip hop's global growth since the 1990s (Lum, 2007), cultivating it for their own cultural identity purposes—sometimes to the surprise of consumers, as reflected upon in the New York Times’ coverage of a breakdance competition: “…once upon a time, Asian‐Americans in hip‐hop would have been treated as a joke, as something cute—like Mr. Rogers popping and locking” (Seibert, 2014). It is essential to acknowledge critiques regarding cultural appropriation by Asian and Asian American artists and performers (Dao, 2014; Reyes, 2005). Hip hop has its origins in Black America and Asian/Asian American accolades for use of hip hop dancing, slang, and other cultural indicators is viewed by some as problematic (Reyes, 2005). Others, however, assert that the widespread popularity of hip hop leads to the invariable outcome that youth globally will participate in hip hop culture and indeed, that hip hop is “at the nucleus of a postmodern web of control over global social narratives of identity” (Osumare, 2009, p. 171). While the debate over Asian youth participation in hip hop is important to continue, contact, assimilation, and marginalization perspectives help explain why hip hop is an appealing identity for SEAA youth.

### Contact Perspective

There is significant variability in socioeconomic status among SEAA immigrant groups but most populations live below the poverty level at higher rates than the U.S. average (11.3% vs. 13% Vietnamese, 18.2% Cambodian, 12.2% Lao and 27.4% Hmong; Southeast Asia Resource Action Center, 2011). As a result, many SEAA families live in low‐income housing or ethnic enclaves with high concentrations of African American and Latinx peers. Due to this proximity and contact, SEAA youth have familiarity with hip hop and share their engagement with the music and art form with their multicultural peers of color in their communities. Moreover, the identification with the Black American experience due to the shared commonality of of marginalization based on ethnic minority status and socioeconomic status is an impetus for turning to hip hop (Basu & Lemelle, 2006; Ramamurthy, 2006).

### Assimilation Perspective

Often perceived as “perpetual foreigners” (Said, 1979), SEAA youth are often in the position of proving their American‐ness to others, including peers within their same ethnic group. They do so by adopting cultural symbols such as dress and language to demarcate identity status (Nguyen & Brown, 2010). Demonstrating their engagement with the most popular form of music may help facilitate others’ impressions of their willingness to adapt to U.S. culture. It is this form of hip hop adoption that may be most problematic, because SEAA use of Black culture for societal acceptance can be viewed as appropriation and leads to strained relationships between Asian and African American communities. Moreover, this engagement with hip hop ignores its history as a sociopolitical voice for the marginalized (Osumare, 2001).

### Marginalization Perspective

When not fighting the perpetual foreigner stereotype, Asian youth are often faced with the challenge of invisibility in a sociocultural landscape that views race along Black and White lines (Wu, 2002). Sharma (2010, p. 3) beautifully articulates the role hip hop plays in overcoming that invisibility for South Asian youth in North America (i.e., “desi”):

…some young South Asians negotiate their racial invisibility in the United States by developing newly racialized identities that express a political consciousness of interminority solidarity…by drawing upon the concept of Blackness, the most visible and salient example of racial identity in the United States. And these desis express their perspectives in the most popular and generationally relevant expression of Blackness at this time—hip hop.

Like their South Asian American counterparts, SEAA youth turn to hip hop to find space on the margins of U.S. society and sometimes, of their home ethnic culture. Indeed, this perspective on SEAA youth participation in hip hop culture arguably reflects the core essence of hip hop, that which binds collective marginalities (Motley & Henderson, 2008).

## Cultural Variability and Hip Hop

It is clear that hip hop serves an important purpose in identity development for SEAA adolescents and emerging adults, particularly in helping them adapt to changing global and local sociocultural contexts. Adolescence (roughly the second decade of life) and emerging adulthood (roughly late teens through twenties) are two developmental periods during which there is an intensification of identity exploration and consolidation, including cultural and ethnic identity development. During this time, there is also is greater access (vs. childhood) and greater openness (vs. later in adulthood) to new non‐familial cultural influences (Arnett, 2000, 2002; Jensen, Arnett, & McKenzie, 2011; Phinney, 1990). A hip hop cultural identity is malleable and can be played up/played down through a process called cultural variability as youth adapt to the sociocultural and interpersonal demands of adolescence/emerging adulthood. *Cultural variability* (CV) is a newly introduced identity development process capturing how youth emphasize or de‐emphasize aspects of a cultural identity, or vary the extent to which that identity has influence over daily behaviors and interactions with others (Ferguson, Nguyen, & Iturbide, 2016). Hip hop provides an ideal cultural identity for the type of dynamic identity play involved in CV because it is uniquely suited for identity flexibility. Indeed, breakdancing—as one element in hip hop culture—is both figurative and literal performance based on identity flexibility and play. For example, within a breakdancing cypher, youth alter their self‐presentation, changing the content of each performance to fit the needs of the local audience (Motley & Henderson, 2008)—much as immigrant youth may utilize CV to adapt their dress or language to better fit the demands of their family or peers (Ferguson et al., 2017).

Mixed methods research with two multicultural emerging adult samples shows that CV is agentically exercised and is context‐driven in both imposed situations (e.g., racism, class assignment; “I chose to de‐emphasize my appearance to avoid seeming unapproachable,” Black participant) and self‐selected situations (e.g., concert, peer interactions; “Today I had a chance and took it to emphasize my cultural identity,” Mexican American participant) (Ferguson et al., 2016). Moreover, CV meets needs related to collective identity and self‐esteem (managing group pride/shame), and acculturation (coping with marginalization, achieving assimilation) (Ferguson et al., 2017). Finally, CV is associated with positive family interactions suggesting that it helps youth accommodate generational differences when interacting with family (e.g., parents vs. siblings). In the remainder of this article, the ways hip hop functions as a cultural identity for SEAA adolescents/emerging adults will be discussed, using qualitative data from a mixed methods study. We examine the significance of hip hop as a glocalized cultural identity, highlighting how SEAA youth utilize CV in this identity in the following ways: (1) as a cultural identity transcendent of race/ethnicity, (2) as a means toward relational and identity harmony, (3) as identity resistance, and (4) as identity belonging.

## New Findings

Participants in this study were 53 SEAA adolescents and emerging adults^1^ (*n* = 23 and 30, respectively; *M*_age_ = 18.57, SD = 1.71) who identify triculturally with Asian, dominant White, and hip hop culture (or Black culture as a proxy for hip hop)^2^. Participants were recruited through multiple means: online via social media, in person through community organizations, at SEAA cultural events (e.g., Hmong New Year), at high school college fairs targeting SEAA youth, and in person at breakdance tournaments. The majority of participants were from the Midwestern United States (Minnesota and Wisconsin) although online recruitment yielded some participants from California. The field research team consisted of three undergraduate RAs and the PI, all of whom identify as Southeast Asian or Asian. The RAs were hip hop cultural participants who themselves breakdance or MC. Potential participants were approached at these events and informed of the study verbally and with flyers; if the setting was appropriate, consent forms were disseminated. Snowball sampling was utilized on social media, where a Facebook event was created for the study and community members who supported the study were invited to tag potential participants through the event.

Local community representatives and members of the hip hop community collaborated with the researchers on the best recruitment techniques in return for monetary compensation to the community organization in the form they desired, such as donations toward dance tournament travel/entry fees paid to event organizers or rental of practice space. We reached out to tournament organizers and dance instructors who introduced our research team on stage at tournaments and validate our presence at the tournaments. In many cases, they were thrilled that a team of SEAA and Asian researchers was interested in their experiences.

Data for this article are drawn from semistructured individual interviews (*n* = 8) conducted by the lead author and thematically analyzed to explore the role of hip hop in cultural identity development. Select excerpts are presented as thematic exemplars across participant responses. Because the research team recruited at breakdance tournaments, several participant observations from the field notes are also reported. However, as this study does not use ethnographic research methodology, the field notes were not collected nor analyzed using the rigorous standards guided by a true ethnographic study.

### Hip Hop Is Transcendent of Race/Ethnicity

Participants viewed hip hop as a cultural identity based on skill as a performer, the contributions one makes to the community, and the shared values of multiculturalism. As a result, it was described as an identity that transcends phenotypic racial and ethnic characteristics, and national/international boundaries:

I think that hip hop has helped me a lot in understanding color within cultures. Because even though you have a united front, which is the dance, the art, the music behind it, the politics that are involved because of the color of your skin can very easily break apart something that was created to bring people together…We've had several conversations with different people, like different dancers here that feel like hip hop is only for black people… But they are not competitors here. They have not earned their merit on the floor… And for us, it's not the color of your skin. It is your merit …Like there's so much more than being a color within it. (Peach, 22)

Because hip hop is multiculturally accepted, it fulfills an identity need that cannot otherwise be fulfilled by their Asian American identity status alone, as John (18) explains:

When I break, I feel like the ethnicity doesn't matter anymore. It's all about the dance. Like the dance becomes like the culture. It becomes the language… Because being Asian, being a minority, sometimes I feel at a disadvantage because…I'm not white, I'm not privileged, you know?…being, at the (University) where it's predominantly white people, the dancing really gives me something to be proud of.

Hip hop forges a common language shared globally which facilitates multicultural communication for SEAA youth. Through hip hop, they can portray themselves as multifaceted, heterogeneous Asian Americans who simultaneously speak to mainstream youth culture while uplifting voices from the margins of the dominant society. Owing to hip hop's global popularity and the relatively positive social standing Asian ethnic group members hold in the global hip hop movement, John and others such as Timothy (19, below) aptly understand that their engagement in hip hop can be played up to convey merit to the dominant culture.

In my opinion, hip hop has always been…well, there's really no term for it. But in my thinking, ethnicity‐neutral. It feels, it actually feels like one of the best feelings in the world. And the reason why is because when I am only devoting myself to hip hop, I can go anywhere, do hip hop, and people can look at it and say, “Oh, that's hip hop.” It doesn't have to be like, “He's Asian,” or, “He's Hmong.” …I do want to say that there are many people of color that feel the way I do as well… It makes me feel better that there are more people *in the world* (emphasis by researcher) that are similar to me and going through the things I'm going through, but yet they still have hip hop and their own identity to fall back on to. And they can just use these things to empower themselves.

The multicultural and racially transcendent nature of hip hop as experienced by participants was felt deeply by the researchers attending breakdance tournaments, in which the dancers, MCs, DJs, judges, and audience were of diverse ethnic, national, age, and cultural backgrounds. Two of the tournaments we attended were hosted by White male dancers who are national breakdance competitors, one of whom is an immigrant from Eastern Europe. The phenotypically Asian and African American judges they brought into the tournaments were introduced as veterans of hip hop (i.e., OGs/“original gangsters”) and were well known nationally. Hip hop, as engaged in by the participants in this study, was both ideologically and observably multicultural and required adept navigation in this physical and psychological space that was not tied to one single ethnocultural group.

### Hip Hop for Relational and Identity Harmony

Hip hop was reported an effective identity for SEAA youth to engage in CV as they played up their hip hop identity for acceptance and greater social status in different peer contexts. However, prior investigations of CV have found that it functions differently in peer and family contexts. If CV is low—that is, when a cultural identity is not played up and down too much each day—SEAA youths’ use of CV for their hip hop identity can facilitate greater acceptance by peers of their own ethnic group and of the dominant culture. But too much engagement in hip hop can run the risk of distancing youth from parents and Asian communities due to negative connotations that hip hop is too closely aligned with either the dominant culture, and therefore viewed as assimilationist, or with Black/African American culture, which begets stereotypes and misperceptions that hip hop is synonymous with gangs, violence, and delinquency (Dao, 2014; Kim, 1999). In this study, there was no evidence to support this supposition.

Participants did not view hip hop as the crucible for their conflict with parents, even when asked explicitly. CV was distinct from identity alternation in that experience such that playing up their hip hop identity did not require playing down their Asian identity. Not only did SEAA youth successfully play up hip hop culture while remaining strongly orientated toward Asian/Asian American culture, but hip hop was described as a source of comfort in parent–child conflicts when participants sought music that resonated with their emotional experiences: “…you're a teenager, you're kind of going through that phase where like your parents don't understand you. I'll listen to music and try to understand my feelings that way” (Aniloc, 20). In this way, hip hop helped to harmonize parent–youth relationships.

Because many SEAA adolescents/emerging adults today have parents who are 1.5‐ or second‐generation immigrants themselves, the Asian American culture is changing. SEAA youth reflexively understand this and adapt by playing up the positive aspects of hip hop culture in order to assuage conflict rather than diminishing either Asian or hip hop culture, as Timothy (19) describes:

…But we're like modern and traditional. It's kind of hard to explain. But we're more modern. My family kind of like understands the kids and they let the kids do what they want to and expect.

*(Okay Because I've heard that in some families for kids who do hip hop, the parents really don't like it. Do you hear of that still happening for some people you know?)*

Yeah, I do, actually. And it's still, I believe it's slowly dying down. But I do hear it and I do hear stories and I have talked with a few friends’ parents about it, trying to promote hip hop in the more positive aspects…It's starting to become rare now, actually…now that like hip hop is being more promoted, then it's becoming more popular. And parents are starting to become more okay with it…

Working toward conflict resolution around hip hop appeared to be a normative aspect of autonomy development that ultimately helped youth integrate their (compatible) Asian/Asian American and hip hop cultural identities:

*(Does the dancer side [of your identity] influence your relationship with your parents at all?)*

It did at one point, because they're just afraid that I would be like so obsessed with dancing that I would just forget my studies and just like be reckless and stuff like that. I feel like my parents, they don't understand passion. Because I don't think that they have one, or they know what their passion is. So sometimes it can clash… (John, 18)

*(Is it fair to say that sometimes it causes a little friction with you and your parents, but it doesn't result in anything, any major outcomes…?)*

No. It fits well together. It's just something that…being Asian American and being a dancer is kind of like this is me and this is what I do. They just fit together. I don't have to switch because I feel that they're just two different things.

The breakdance tournaments our research team attended were observed to have parents and families (including young children/siblings) in the audience and there appeared to be significant community support for the events, supporting participant reports regarding lack of parental conflict over hip hop.

Findings are consistent with, and help to explain, prior CV findings in multicultural samples indicating that CV is associated with more positive family interactions (Ferguson et al., 2017). The changing nature of SEAA parent–child relationships should be part of our evolving understanding of dynamic SEAA adolescent/emerging adult identities. Parents are likely socializing their children toward cultural norms that are well beyond the binary of dominant versus traditional/ethnic culture and seem to no longer view youth engagement in hip hop as an assimilative loss of “traditional” Asian culture. Rather, hip hop engagement seems to help harmonize parent–youth relations and as a consequence, helps to foster cultural identity integration (i.e., Benet‐Martínez & Haritatos, 2005).

### Hip Hop as Identity Resistance

As previously stated, hip hop originated as a source of empowerment and resistance against the societal mainstream that perpetuates discrimination and racism of ethnic minority population. Ethnic minority youth, including SEAA adolescents/emerging adults, “play up” hip hop identity in the face of racism and obstruct stereotyped thinking about their ethnic group (Dao, 2014; Pulido, 2009). Two paradoxical categories of stereotypes against SEAA youth persist: that they are engaged in negative behaviors (i.e., gangsters) or that they are model minorities. In schools and communities with high numbers of SEAA second‐generation youth, negative stereotypes persist that they are delinquent, apathetic, and inclined toward violence or early pregnancy, and other problematic behaviors that youth must continually combat (Lee, 2001; Chhuon, 2014).

In contrast, Asian Americans in the United States are often rhetorically elevated as “model immigrants” as the baseline for immigrant and ethnic minority success, without regard for the wide variance of “success” within the Asian American population and the institutional and systemic barriers that prevent successful outcomes for all ethnic minority groups. When data are disaggregated, many SEAA groups experience poor economic, socioemotional, and academic outcomes—a sharp departure from East Asian and South Asian groups (Equitable Growth, 2016; Ngo & Lee, 2007; Yang, 2004). Therefore, by aligning themselves with an identity closely associated with Black culture and emphasizing that identity through CV, SEAA youth implicitly—and sometimes explicitly—resist the model minority stereotype, which is used to sustain anti‐Black racism and White supremacy in the United States and globally (Poon et al., 2015).

SEAA youth turn to hip hop both to contest these stereotypes and to elevate the positive profile of Asian American and SEAA populations: “I am a Hmong person who is dancing, who is doing hip hop. We are not that typical stereotype where we are involved with gangs and drugs and alcohol and all that stuff. So I use it kind of as an empowerment as well.” (Timothy, 19).

By playing up their hip hop cultural identity, SEAA youth assert agency over crafting their own identities and narrative about that identity—sometimes literally finding their voice through rap and the language of hip hop:

During like 1990s or ‘80s, we grew up with strict Asian parents who came from the motherland. So traditions would fall upon us really hard, and we feel like we don't have a voice… We try to talk to our parents. We ask them questions, but the strict parents thought we were talking back. Hip hop comes in, because they let anyone speak their minds. Whether it's bad lyrics, or very meaningful lyrics or movements or whatever… Everyone is more, everyone's respectful to them. You show us who you are. We respect that. That's how Asian people, teenagers, my age group, were drawn towards it. (Danny, 22)

As SEAA youth found their voice in hop hip, they become engaged in the cultural movement. Asian youth have turned to numerous subcultures to resist marginalization and counter assumptions others make about their group, including the “club” (i.e., dance/drug) subculture of the late 1990s (Hunt, Moloney, & Evans, 2011) or the import car subculture (Kwon, 2004). However, hip hop is unique in it's requirement that participants actively contribute to defining and shaping the culture. And today, hip hop's cultural artifacts are shared widely online, enlarging the global cypher to which SEAA youth contribute, uniting with marginalized individuals from around the world who are challenging racism and advocating for immigrant rights through hip hop arts (see review in Dao, 2014).

### Hip Hop as Belonging

As SEAA youth create content that contributes to, reinterprets, and defines hip hop culture (Morgan & Bennett, 2011), they forge their own niche within the local sociocultural context and global movement of hip hop society. Locally, participants report that the majority of their hip hop cultural peers share the same diasporic Asian identity as they do, yet also identify on the margins of their home and dominant culture. Therefore, a hybrid identity of hip hop is an important source of identity integration for the participants, including Lizzie (22):

I would say that hip hop helps me be among people who are more like me, like physically and culturally. I started hip hop in high school, the beginning of high school. And before that, I felt more Cambodian, because I was still learning to become fluent in English and… I felt like I was still discovering American culture. And doing that (hip hop), I felt more connected to my school, to everyone at the school, no matter what ethnicity they were. And I felt like we all shared that in common. We all liked hip hop. And I felt like among people who are Cambodian… I felt at home because I was among Southeast Asian people, for sure. I felt like more myself. *So it is a bridge*. [emphasis by researcher]

Hip hop is therefore a fluid, hybrid identity that allows SEAA youth to engage in multiple cultural worlds simultaneously.

## Conclusion

Hip hop is a global phenomenon that offers one pathway for Southeast Asian immigrant adolescents in the United States to achieve an integrated cultural identity. The popularity of hip hop culture elevates local acceptance of SEAA youth and facilitates their “glocal” sense of belonging (Barrett, 2012). SEAA youth navigation between the three cultures of Asian, dominant White, and hip hop has implications for how we think about tricultural identity development. Whereas ethnoracial “triangulation” has been discussed in sociological discourse on Asian American youth (Kim, 1999), tricultural identities require greater attention in developmental science, with focus on hybrid identities that help bridge multiple aspects of an individual's sense of self: ethnic, social, and political (Ferguson et al., 2012; Medina, in press; Nguyen, 2013). Ethnic minority and immigrant adolescents/emerging adults creatively and agentically craft identities that transcend the ethnic and racial classifications traditionally available to them (i.e., census categories) by using identities such as hip hop that are hybridized, multifaceted, and drawn from multiple cultures to better capture their sense of self, provide belonging, and allow them to make meaningful contributions to society.

In their important piece on globalization and cultural identity, Jensen et al. (2011) call for greater attention to globalization and youth civic engagement. Exploration of new cultural identities such as hip hop contribute to our understanding of youth sociopolitical engagement. As youth participate in hip hop's global cypher, they contribute to voices of resistance against personal and institutional racism, discrimination, and stereotypes that impact their lives and the lives of ethnic minority people around the globe. Hip hop is uniquely suited for civic engagement. Community organizers and educators use it to develop critical consciousness and build confidence in public performance that empowers youth to speak out about local and global issues of importance to them (Clay, 2012; Ginwright, Noguera, & Cammarota, 2006)—as long as the core tenets of hip hop as youth resistance are retained.

In turn, as SEAA youth participate in hip hop culture, the culture recursively informs their own identities. To “remix” is a core activity in hip hop: combining existing materials to produce new forms of music. Remix culture itself is “the global activity consisting of the creative and efficient exchange of information made possible by digital technologies” (Navas, n.d.). Drawing from remix theory, the cultural identity development of SEAA youth and their tricultural peers can be viewed as an ongoing process in which the existing cultural practices inform identity development, but are then agentically and dynamically played with locally and globally to create a new cultural medium that will in turn, inform new identities.

An evolving global society requires new concepts and frameworks for understanding identity development including cultural variability (CV). CV helps us understand how tricultural identities may be negotiated real time through flexible identity play and adjustments to one or more cultural identities simultaneously. SEAA youth deployment of their hip hop cultural identities is a clear exemplar of CV in action. Identity development processes often emphasize commitment to, and pride in, an ethnic or cultural identity. But playing up hip hop identity to emphasize distance from other cultural groups opens up an intriguing possibility that CV processes also help youth affirm who they are *not*. The ways CV is used as identity resistance warrants further exploration.

Through sharing hip hop performance, crafting of shared language (Alim & Pennycook, 2007), and engagement in social justice glocally, SEAA youth simultaneously internalize and influence the global culture. Participation in hip hop's global cypher allows SEAA youth and others worldwide to express who they are and to resist the mainstream narratives, stereotypes, and misconceptions of their identities while carving a place of belonging in an ever‐expanding global society.

## Notes

## Footnotes

1 The combined sample of adolescents (age 17 years and under) and emerging adults (ages 18–22) will be referred to as “youth” henceforth unless referring to a specific developmental stage.

2 We acknowledge the political discussion around (de‐)capitalization of Black and White racialized identities (e.g., note in Crenshaw, 1991; Perlman, 2015) and have chosen to capitalize all ethnocultural identities because they are discussed herein as specific cultural identities rather than racial identities.
